# Supplementary material for: A Longitudinal Study of Chronic Periodontitis in Two Cohorts of Community-Dwelling Elderly Australians
Source: Int J Environ Res Public Health. 2022 Sep 19;19(18):11824. doi: 10.3390/ijerph191811824 (PMC9516963; doi:10.3390/ijerph191811824)
Supplement: Supplementary file 1 [file ijerph-19-11824-s001.zip › ijerph-1850666-supplementary.pdf]

Supplemental tables

Table S1: Baseline characteristics of dentate Australian older adults who were examined at 2 years compared with those who were lost to follow-up.

	SADLS I			SADLS II		
	Examined	Lost to follow-up	P-value	Examined	Lost to follow-up	P-value
	N (%)	N (%)		N (%)	N (%)	
**Total**	567	234		201	154	
Sample demographic characteristics						
**Age groups**			**0.0163**			0.3364
60-64	157 (27.7)	49 (20.9)		54 (27.4)	53 (29.4)	
65-69	153 (27.0)	58 (24.8)		65 (33.0)	54 (30.0)	
70-74	94 (16.5)	39 (16.7)		41 (20.8)	26 (14.4)	
75-79	101 (17.8)	47 (20.1)		21 (10.7)	25 (13.9)	
80-84	49 (8.6)	25 (10.7)		12 (6.1)	19 (10.6)	
≥ 85	13 (2.3)	16 (6.8)		4 (2.0)	3 (1.7)	
**Sex**			0.8825			0.1685
Female	227 (40.0)	95 (40.6)		91 (46.0)	96 (53.0)	
Male	340 (60.0)	139 (59.4)		107 (54.0)	85 (47.0)	
**Married status**			0.1895			0.9734
Married/ De-facto	420 (74.1)	162 (69.5)		148 (75.1)	137 (75.3)	
Single	147 (25.9)	71 (30.5)		49 (24.9)	45 (24.7)	
**Born in Australia**			0.9063			**0.0108**
Yes	387 (68.4)	159 (68.0)		143 (74.5)	110 (62.2)	
No	179 (31.6)	75 (32.1)		49 (25.5)	67 (37.9)	
**Education level**			0.5394			0.3048
Tertiary	193 (34.1)	81 (34.6)		36 (18.3)	39 (21.4)	
Trade/ diploma degree	113 (20.0)	39 (16.7)		61 (31.0)	65 (35.7)	
Secondary	260 (45.9)	114 (48.7)		100 (50.8)	78 (42.9)	
**Household income**			0.0516			0.6866
High	222 (41.4)	70 (32.3)		64 (37.4)	63 (41.7)	
Medium	177 (33.0)	78 (35.9)		49 (28.7)	38 25.2)	
Low	137 (25.6)	69 (31.8)		58 (33.9)	50 (33.1)	
**Oral health related behaviours**						
**Dental insured**			0.1241			0.2388
Had	244 (43.4)	87 (37.5)		126 (63.3)	127 (69.0)	
No	318 (56.6)	145 (62.5)		73 (36.7)	57 (31.0)	
**Smoke status**			0.9340			0.6995
Never smoked	252 (44.9)	108(46.4)		116 (59.2)	99 (55.9)	
Used smoker	245 (43.7)	99 (42.5)		72 (36.7)	68 (38.4)	
Current smoker	64 (11.4)	26 (11.2)		8 (4.1)	10 (5.7)	
**Alcohol drinking**			0.1734			0.4677
No	136 (24.0)	67 (28.6)		36 (18.3)	28 (15.5)	
Yes	430 (76.0)	167 (71.4)		161 (81.7)	153 (84.5)	
**Dental Behaviours**						
**Oral hygiene (Tooth brushing)**			0.0548			0.9733
At least twice/day	361 (63.7)	132 (56.4)		138 (69.4)	128 (69.2)	
Less than twice/day	206 (36.3)	102 (36.3)		61 (30.7)	57 (30.8)	
**Last dental visiting**			**0.0070**			0.4921
Less than 12 months	360 (63.7)	124 (53.5)		139 (70.2)	135 (73.4)	
More than 12 months	205 (36.3)	108 (46.6)		59 (29.8)	49 (26.6)	
**Reasons for dental visiting**			**0.0065**			0.2839
Check	252 (44.7)	79 (34.2)		89 (46.1)	94 (51.7)	
Problem	312 (55.3)	152 (65.8)		104 (53.9)	88 (48.4)	

Note: P-value: Chi-square test (or Fisher's exact test for cell values less than 5).

Table S2: Baseline general health condition of dentate Australian older adults who were examined at 2 years compared with those who were lost to follow-up.

	SADLS I			SADLS II		
	Examined	Lost to follow-up	P-value	Examined	Lost to follow-up	P-value
**Total**						
**General health**						
**Asthma**			0.7296			0.2185
No	525 (92.6)	215 (91.9)		165 (86.8)	143 (82.2)	
Yes	42 (68.9)	19 (8.1)		25 (13.2)	31 (17.8)	
**Arthritis**			0.5614			0.8102
No	280 (49.5)	110 (47.2)		97 (51.9)	85 (50.6)	
Yes	286 (50.5)	123 (52.8)		90 (48.1)	83 (49.4)	
**Cancer**			0.2428			0.2323
No	502 (88.9)	201 (85.9)		159 (84.6)	138 (79.8)	
Yes	63 (11.2)	33 (14.1)		29 (15.4)	35 (20.2)	
**Cataracts**			0.9072			0.2187
No	472 (83.3)	194 (82.9)		147 (80.3)	125 (74.9)	
Yes	95 (16.8)	40 (17.1)		36 (19.7)	42 (25.2)	
**COPD**			0.0328			0.2471
No	505 (89.2)	196 (83.8)		167 (90.8)	158 (94.1)	
Yes	61 (10.8)	38 (16.2)		17 (9.2)	10 (6.0)	
**Diabetes**			0.0017			0.4244
No	542 (95.6)	210 (89.7)		161 (85.2)	142 (82.1)	
Yes	25 (4.4)	24 (10.3)		28 (14.8)	31 (17.9)	
**Hypertension**			0.6984			0.3984
No	365 (64.4)	154 (65.8)		94 (49.5)	77 (45.0)	
Yes	202 (35.6)	80 (34.2)		96 (50.5)	94 (55.0)	
**Heart**			0.1382			0.2560
No	465 (82.3)	182 (77.8)		170 (91.9)	151 (88.3)	
Yes	100 (17.7)	52 (22.2)		15 (8.1)	20 (11.7)	
**Osteoporosis or hip fracture**			0.1182			0.4105
No	546 (96.5)	220 (94.0)		168 (89.8)	148 (87.1)	
Yes	20 (3.5)	14 (6.0)		19 (10.2)	22 (12.9)	
**Stroke**			0.3070			0.1282
No	529 (93.5)	213 (91.4)		182 (97.3)	159 (94.1)	
Yes	37 (64.9)	20 (8.6)		5 (2.7)	10 (5.9)	
**Chronic diseases (At least one)**			0.0090			0.7604
No	123 (21.7)	32 (13.7)		29 (14.7)	29 (15.9)	
Had	444 (78.3)	202 (86.3)		168 (85.3)	154 (84.2)	
**Number of diseases**			0.0679			0.2075
0	123 (21.7)	32 (13.7)		29 (14.7)	29 (15.9)	
1	163 (28.8)	78 33.3)		62 (31.5)	44 24.0)	
2	151 (26.6)	53 (22.7)		53 26.9)	47 (25.7)	
3	79 (13.9)	39 16.7)		30 (15.2)	29 (15.9)	
4	34 6.0)	21 (9.0)		15 (7.6)	19 (10.4)	
5	10 (1.8)	8 (3.4)		6 (3.1)	14 (7.7)	
6	6 (1.1)	3 (1.3)		2 (1.0)	0 (0.0)	
7	1 (0.2)	0 (0.0)		0 (0.0)	1 (0.6)	

Note: P-value: Chi-square test (or Fisher's exact test for cell values less than 5).

Table S3: Prevalence of periodontitis and mean number of tooth loss of Australian adults aged 60+ years in two surveys.

	**SADLS I** (n=567)		**SADLS II** (n=201)	
	Baseline (1991-92)	2 years follow-up	Baseline (2013-14)	2 years follow-up
	N (%)	N (%)	N (%)	N (%)
**Degree of periodontitis**				
**AAP-CDC-case**				
No/Mild	*154 (27.2)	*226 (39.9)	*40 (19.9)	*68 (33.8)
Moderate	342 (60.3)	292 (51.5)	127 (63.2)	119 (59.2)
Severe	71 (12.5)	49 (8.6)	34 (16.9)	14 (7.0)
**EFP/AAP case**				
Stage I	*31 (5.5)	*66 (11.6)	*6 (3.5)	*22 (11.0)
Stage II	295 (52.0)	313 (55.2)	74 (43.5)	113 (56.2)
Stage III-IV	241 (42.5)	188 (33.2)	90 (52.9)	66 (32.8)
	Mean ([a] SD)	Mean (SD)	Mean (SD)	Mean (SD)
**No. of tooth loss**	12.5 (6.7)	12.8 (6.8)	6.3 (6.0)	6.6 (6.0)

Notes: *Chi-square test, and p-values <0.05; [a] SD: standard deviation.

Table S4: Multivariable models of the incidence rate ratios (IRRs) of periodontitis under two case definition in **SADLSI** survey.

	CDC/AAP			EFP/AAP		
	**Model 1**	**Model 2**	**Model3**	**Model 1**	**Model 2**	**Model3**
Sample demographic characteristics						
**Age groups**						
60-64	ref	ref	ref	ref	ref	ref
65-69	0.73 (0.44-1.22)	0.69 (0.41-1.17)	0.68 (0.39-1.16)	1.08 (0.64-1.82)	1.08 (0.64-1.85)	1.09 (0.63-1.88)
70-74	0.83 (0.47-1.46)	0.82 (0.46-1.47)	0.72 (0.41-1.31)	1.09 (0.60-1.98)	1.00 (0.54-1.87)	0.95 (0.51-1.78)
75-79	0.83 (0.47-1.44)	0.90 (0.51-1.59)	0.75 (0.41-1.38)	1.14 (0.64-2.03)	1.17 (0.64-2.14)	1.06 (0.56-2.00)
80-84	0.84 (0.41-1.72)	0.86 (0.41-1.81)	0.74 (0.34-1.59)	1.09 (0.52-2.31)	1.10 (0.51-2.36)	1.02 (0.47-2.23)
≥ 85	1.01 90.38-3.20)	1.16 (0.40-3.38)	0.87 (0.29-2.65)	1.01 (0.30-3.41)	1.08 (0.32-3.68)	1.00 (0.28-3.54)
**Sex**						
Female	ref	ref	ref	ref	ref	ref
Male	**1.81 (1.16-2.84)**	1.38 (0.84-2.29)	1.25 (0.75-2.10)	**1.51 (1.00-2.35)**	1.31 (0.79-2.19)	1.26 (0.75-2.12)
**Married status**						
Married/ De-facto	ref	ref	ref	ref	ref	ref
Single	1.00 (0.58-1.72)	0.94 (0.54-1.62)	1.00 (0.57-1.74)	1.13 (0.66-1.91)	1.08 (0.63-1.86)	1.13 (0.65-1.95)
**Born in Australia**						
Yes	ref	ref	ref	ref	ref	ref
No	1.29 (0.88-1.90)	1.32 (0.90-1.96)	1.41 (0.94-2.12)	1.32 (0.89-1.95)	1.29 (0.86-1.93)	1.31 (0.87-1.98)
**Education level**						
Tertiary	ref	ref	ref	ref	ref	ref
Trade/ diploma degree	0.80 (0.47-1.37)	0.79 (0.46-1.37)	0.85 (0.49-1.49)	0.64 (0.35-1.15)	0.62 (0.34-1.14)	0.68 (0.37-1.25)
Secondary	0.82 (0.54-1.27)	0.87 (0.55-1.35)	0.88 (0.55-1.38)	0.90 (0.59-1.39)	0.96 (0.61-1.50)	0.92 (0.58-1.47)
**Household income**						
High	ref	ref	ref	ref	ref	ref
Medium	1.31 (0.85-2.02)	1.34 (0.83-2.17)	1.32 (0.81-2.15)	1.20 (0.77-1.88)	1.17 (0.71-1.93)	1.21 (0.73-2.01)
Low	1.13 (0.65-1.94)	1.19 (0.66-2.14)	1.04 (0.57-1.92)	1.07 (0.62-1.84)	1.05 (0.58-1.91)	0.99 (0.53-1.83)
**Oral health and related behaviours**						
**Dental insured**						
Had		ref	ref		ref	ref
No		0.99 (0.63-1.56)	1.06 (0.67-1.68)		1.10 (0.69-1.76)	1.14 (0.71-1.82)
**Smoke status**						
Never smoked		ref	ref		ref	ref
Used smoker		1.52 (0.95-2.44)	1.59 (0.98-2.58)		1.38 (0.86-2.21)	1.56 (0.92-2.45)
Current smoker		**2.32 (1.29-4.18)**	**2.38 (1.30-4.35)**		**2.10 (1.15-3.85)**	**2.30 (1.24-4.26)**
**Alcohol drinking**						
No		ref	ref		ref	ref
Yes		1.20 (0.72-2.01)	1.26 (0.75-2.14)		0.93 (0.57-1.52)	0.90 (0.54-1.50)
**Oral hygiene (Tooth brushing)**						
At least twice/day		ref	ref		ref	ref

Less than twice/day	0.97 (0.65-1.44)	0.96 (0.63-1.46)	0.97 (0.64-1.46)	0.95 (0.62-1.46)
**Last dental visiting**				
Less than 12 months	ref	ref	ref	ref
More than 12 months	0.81 (0.50-1.30)	0.71 (0.43-1.16)	0.71 (0.44-1.16)	0.69 (0.42-1.14)
**Reasons for dental visiting**				
Check	ref	ref	ref	ref
Problem	1.05 (0.66-1.67)	1.15 (0.71-1.83)	1.13 (0.70-1.80)	1.13 (0.70-1.83)
**General health**				
**Asthma**				
No		ref		ref
Yes		1.39 (0.73-2.66)		1.28 (0.65-2.52)
**Arthritis**				
No		ref		ref
Yes		1.10 (0.74-2.31)		1.28 (0.85-1.95)
**Cancer**				
No		ref		ref
Yes		1.30 (0.73-2.31)		1.38 (0.77-2.44)
**Cataracts**				
No		ref		ref
Yes		1.37 (0.83-2.28)		0.87 (0.49-1.55)
**COPD**				
No		ref		ref
Yes		0.66 (0.33-1.32)		0.92 (0.47-1.78)
**Diabetes**				
No		ref		ref
Yes		1.56 (0.69-3.51)		0.96 (0.34-2.72)
**Hypertension**				
No		ref		ref
Yes		0.95 (0.61-3.51)		0.89 (0.57-1.41)
**Heart**				
No		ref		ref
Yes		1.11 (0.68-1.79)		1.18 (0.71-1.96)
**Osteoporosis or hip fracture**				
No		ref		ref
Yes		0.29 (0.03-2.11)		0.29 (0.04-2.12)
**Stroke**				
No		ref		ref
Yes		1.38 (0.69-2.73)		0.88 (0.39-2.00)

Note: Bold used for the statistically significant.

Table S5: Multivariable models of the incidence rate ratios (IRRs) of periodontitis under two case definition in **SADLSII** survey.

	CDC/AAP			EFP/AAP		
	**Model 1**	**Model 2**	**Model3**	**Model 1**	**Model 2**	**Model3**
**Sample demographic characteristics**						
**Age groups**						
60-64	ref	ref	ref	ref	ref	ref
65-69	1.00 (0.56-1.80)	1.06 (0.69-1.64)	1.02 (0.48-2.17)	1.18 (0.62-2.22)	1.09 (0.69-1.72)	1.27 (0.55-2.96)
70-74	0.51 (0.22-1.16)	0.61 (0.33-1.14)	**0.29 (0.09-0.98)**	0.73 (0.32-1.67)	0.80 (0.45-1.41)	0.48 (0.15-1.58)
75-79	1.16 (0.51-2.63)	1.34 (0.68-2.63)	1.30 (0.43-3.90)	1.64 (0.72-3.73)	1.48 (0.76-2.87)	2.23 (0.71-6.34)
80-84	0.91 (0.30-2.76)	0.86 (0.35-2.11)	0.73 (0.18-2.92)	1.13 (0.37-3.52)	0.86 (0.37-1.98)	0.78 (0.18-3.39)
≥ 85	0.62 (0.08-4.82)	0.75 (0.19-2.97)	0.72 (0.08-6.53)	0.74 (0.09-5.85)	0.90 (0.24-3.35)	0.76 (0.08-7.55)
**Sex**						
Female	ref	ref	ref	ref	ref	ref
Male	0.78 (0.48-1.29)	0.92 (0.60-1.41)	0.96 (0.50-1.86)	0.76 (0.45-1.27)	0.87 (0.58-1.31)	0.67 (0.33-1.34)
**Married status**						
Married/ De-facto	ref	ref	ref	ref	ref	ref
Single	0.79 (0.44-1.45)	0.77 (0.47-1.27)	0.67 (0.29-1.51)	0.96 (0.53-1.73)	0.95 (0.61-1.50)	0.78 (0.34-1.78)
**Born in Australia**						
Yes	ref	ref	ref	ref	ref	ref
No	0.94 (0.54-1.66)	0.88 (0.55-1.41)	0.78 (0.35-1.76)	1.01 (0.57-1.79)	1.04 (0.67-1.61)	0.81 (0.35-1.90)
**Education level**						
Tertiary	ref	ref	ref	ref	ref	ref
Trade/ diploma degree	0.74 (0.38-1.42)	0.68 (0.37-1.25)	0.61 (0.27-1.38)	0.76 (0.39-1.46)	0.71 (0.41-1.26)	0.69 (0.30-1.58)
Secondary	0.68 (0.36-1.32)	0.71 (0.38-1.30)	0.67 (0.30-1.49)	0.54 (0.28-1.07)	0.59 (0.32-1.08)	0.48 (0.20-1.14)
**Household income**						
High	ref	ref	ref	ref	ref	ref
Medium	1.08 (0.57-2.02)	0.88 (0.53-1.47)	0.67 (0.28-1.64)	1.20 (0.63-2.30)	1.02 (0.63-1.67)	0.86 (0.34-2.16)
Low	1.19 (0.63-2.25)	1.03 (0.62-1.73)	1.13 (0.52-2.48)	1.21 (0.63-2.34)	1.10 (0.66-1.81)	1.39 (0.61-3.14)
**Oral health and related behaviours**						
**Dental insured**						
Had		ref	ref		ref	ref
No		1.23 (0.78-1.94)	1.42 (0.63-3.18)		1.16 (0.73-1.84)	1.42 (0.59-3.39)
**Smoke status**						
Never smoked		ref	ref		ref	ref
Used smoker		0.77 (0.50-1.19)	0.76 (0.39-1.46)		0.72 (0.47-1.10)	0.49 (0.24-1.02)
Current smoker		1.33 (0.55-3.22)	1.17 (0.23-5.94)		0.95 (0.42-2.16)	0.40 (0.05-3.59)
**Alcohol drinking**						
No		ref	ref		ref	ref
Yes		1.01 (0.58-1.76)	0.78 (0.28-2.22)		1.03 (0.62-1.70)	0.89 (0.32-2.50)
**Oral hygiene (Tooth brushing)**						
At least twice/day		ref	ref		ref	ref

Less than twice/day	1.29 90.83-2.01)	1.40 (0.75-2.61)		1.33 (0.86-2.06)	1.49 (0.78-2.84)
**Last dental visiting**					
Less than 12 months	ref	ref		ref	ref
More than 12 months	1.09 (0.68-1.75)	1.08 (0.48-2.42)		0.84 (0.52-1.37)	0.68 (0.28-1.66)
**Reasons for dental visiting**					
Check	ref	ref		ref	ref
Problem	0.92 (0.61-1.38)	0.93 (0.47-1.87)		1.07 (0.70-1.64)	1.17 (0.58-2.39)
**General health**					
**Asthma**					
No		ref			ref
Yes		1.15 (0.46-2.85)			1.46 (0.57-3.75)
**Arthritis**					
No		ref			ref
Yes		0.93 (0.48-1.80)			0.81 (0.41-1.60)
**Cancer**					
No		ref			ref
Yes		1.68 (0.77-3.67)			1.52 (0.66-3.48)
**Cataracts**					
No		ref			ref
Yes		0.91 (0.37-2.22)			0.92 (0.36-2.35)
**COPD**					
No		ref			ref
Yes		1.14 (0.31-4.27)			1.39 (0.36-5.36)
**Diabetes**					
No		ref			ref
Yes		0.55 (0.21-1.44)			0.30 (0.09-1.08)
**Hypertension**					
No		ref			ref
Yes		1.01 (0.53-1.92)			1.28 (0.65-2.53)
**Heart**					
No		ref			ref
Yes		0.49 (0.13-1.92)			1.05 (0.30-3.66)
**Osteoporosis or hip fracture**					
No		ref	ref	ref	ref
Yes		0.84 (0.28-2.49)			0.42 (0.11-1.59)
**Stroke**					
No		ref	ref	ref	ref
Yes		1.20 (0.10-13.7)			0.83 (0.07-9.31)
